# Water-Light Interaction and Its Effect on the Morphophysiology of *Cedrela fissilis* Vell. Seedlings

**DOI:** 10.3390/plants13182654

**Published:** 2024-09-22

**Authors:** Juliana Milene Silverio, Silvana de Paula Quintão Scalon, Cleberton Correia Santos, Jéssica Aline Linné, Anderson dos Santos Dias, Rodrigo da Silva Bernardes, Thaise Dantas

**Affiliations:** Faculty of Agricultural Science, Federal University of Grande Dourados, Road Dourados-Itahum Km 1, Dourados 79804970, MS, Brazil; juliana.milene@hotmail.com (J.M.S.); cleber_frs@yahoo.com.br (C.C.S.); jessica.aline.linne@gmail.com (J.A.L.); andersondias.agro@outlook.com (A.d.S.D.); rodrigo.bernardes95@hotmail.com (R.d.S.B.); thaise-dantas@hotmail.com (T.D.)

**Keywords:** abiotic stress, cedar, phenotypic plasticity, photosynthetic metabolism

## Abstract

Plant responses to different light and water availability are variable among species and their respective phenotypic plasticity, and the combination between these two abiotic factors can alleviate or intensify stressful effects. This study aimed to evaluate the impacts of exposure time of *Cedrela fissilis* Vell. seedlings to different water and light availability considering natural radiation variations and the interaction of these factors. Seedlings were submitted to combinations of three shading levels—SH (0, 30 and 70%) and three water regimes based on the water holding capacity (WHC) in the substrate, constituting nine cultivation conditions: T1—0% SH + 40% WHC; T2—0% SH + 70% WHC; T3—0% SH + 100% WHC; T4—30% SH + 40% WHC; T5—30% SH + 70% WHC; T6—30% SH + 100% WHC; T7—70% SH + 40% WHC; T8—70% SH + 70% WHC; T9—70% SH + 100% WHC. *C. fissilis* seedlings are sensitive to water deficit, here represented by 40% WHC, regardless of exposure time, and when cultivated in full sun even though there are variations in radiation, the stressful effects were enhanced, acting in a synergistic manner. The condition that provided better gas exchange performance and greater total dry mass accumulation for *C. fissilis* seedlings was 30% shading combined with 100% WHC. *C. fissilis* seedlings have physiological plasticity and resilience to survive under different water and light conditions.

## 1. Introduction

In their initial development phase, especially when they are in the seedling phase, plants are highly responsive to the abiotic factors of the environment in which they are inserted or produced [1]. These factors fluctuate in a marked way due to global climate changes that have occurred in recent years [2]. Decreased precipitation, changes in incident light, increased evapotranspiration, and increased air temperature are some factors that can influence plant growth, development, and performance. It is important to understand the interactions between environmental changes and plant species to implement strategies that drive a future agenda for the agroforestry system, boosting forest recovery and restoration [3,4].

Afforestation and reforestation of areas have increased and consequently seedling production, and therefore the cultivation conditions must be known to obtain good-quality seedlings. To achieve this objective, knowledge of the water and light requirements of each species and its adjustment potential through phenotypic plasticity is essential, which is the plant’s ability to deal with environmental stresses by changing its characteristics. Through adjustments in photosynthetic biochemistry, leaf anatomy, and morphology, plants have the ability to acclimatize to different light and water availability, which can guarantee their survival and improve their growth [5,6].

Light is essential for plant growth and development, which regulates its physiology throughout its cycle; however, it is necessary to understand the ecological classification of each species as either too little or too much light exposure can lead to a stressful cultivation condition, especially at the initial phase. As water availability, especially water deficit, is also a limiting factor for the establishment and development of forest species, plants respond to low soil water content through a complex chain of mechanisms that constitute their adaptation strategies [7].

Each species responds in a different way when submitted to different light and water availability, and depending on the interaction between these two factors, it can alleviate stressful effects [8,9]. These responses are important, as they define species with desirable characteristics for forest restoration and classify those that need shading to develop in open areas and even species that require shading and regular irrigation if used in reforestation. The usefulness of a given species is determined by its tolerance to stressors at the initial stages of its life cycle [10].

*Cedrela fissilis* Vell. (Meliaceae) is among native species with potential for restoration of degraded areas and enrichment plantations, which prefers clayey, well-drained soils [11,12]. This species has high ecological and economic value and has been gaining prominence in the national and international forestry sector, being recommended for landscaping and urban afforestation, among others. These characteristics led to predatory logging, which resulted in the species currently being classified as vulnerable to extinction [13].

However, there are divergences regarding the ecological classification of this species, as some authors classify it as a pioneer species [14], while others classify it as an initial secondary species, depending on the stage of development [15], in which case excess shade can be stressful for *C. fissilis*, and understanding its mechanism when submitted to different light levels and which of these conditions can be stressful or can mitigate the deficit is important.

The present study aimed to understand the effects of exposure time to different water and light availability considering natural radiation variations and the interaction of these factors on gas exchange and the initial growth of *Cedrela fissilis* Vell. seedlings. Given the above, the following questions were proposed: (i) Is water deficit stressful for *C. fissilis* seedlings, regardless of exposure time? (ii) As a pioneer species, can excess shade harm the development of *C. fissilis* seedlings? (iii) Does shading accentuate the effects of water deficit on the photosynthetic and growth responses of *C. fissilis*? (iv) Does this species show resilience and plasticity to adapt to different light and water conditions?

## 2. Results

### 2.1. Visual Aspects

**Figure 1:**
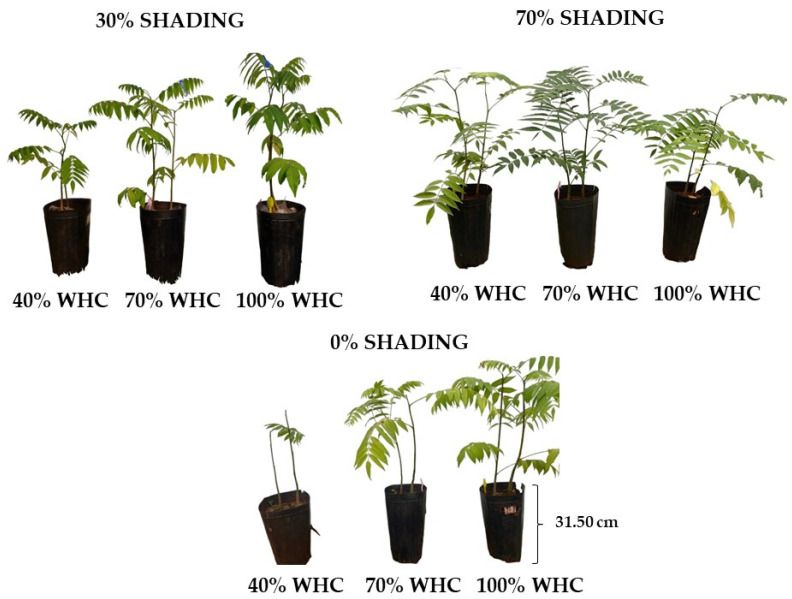
Visual aspects of *Cedrela fissilis* Vell. seedlings of cultivation under different water and light availability. WHC—water holding capacity.

*Cedrela fissilis* Vell. seedlings were responsive to different shading levels and water regimes, in which those grown in 30% and 70% SH with 40% WHC presented better visual appearance than those grown in 0% SH in the same water regime (Figure 1). Furthermore, it is noteworthy that as water availability increased under 0 and 30% SH, seedlings showed greater height and number of leaves, while seedlings under 70% SH had greater uniformity regardless of water regime.

### 2.2. Gas Exchange and Chlorophyll Index

**Figure 2:**
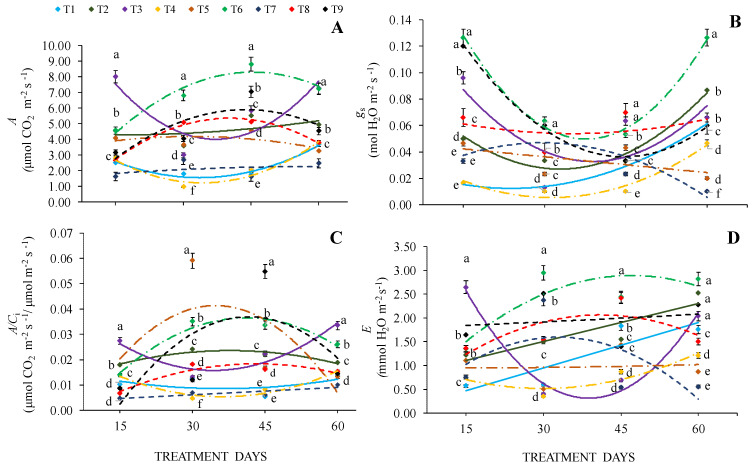
Net photosynthetic rate—*A* (**A**), stomatal conductance—*g*_s_ (**B**), Rubisco carboxylation efficiencies—*A/C*_i_ (**C**), and transpiration rate—*E* (**D**) of *Cedrela fissilis* Vell. seedlings evaluated at 15, 30, 45, and 60 treatment days under different water and light availability. T1—0% SH + 40% WHC; T2—0% SH + 70% WHC; T3—0% SH + 100% WHC; T4—30% SH + 40% WHC; T5—30% SH + 70% WHC; T6—30% SH + 100% WHC; T7—70% SH + 40% WHC; T8—70% SH + 70% WHC; T9—70% SH + 100% WHC. SH—shading and WHC—water holding capacity. Equal lowercase letters between markers of different colors in each evaluation period do not differ statistically by the Scott-Knott test (*p* ≤ 0.05) ± standard error for cultivation conditions.

*Cedrela fissilis* Vell. seedlings grown under 0% SH + 100% WHC and 30% SH + 100% WHC conditions showed the highest *A* values (8.0 and 8.8 μmol CO_2_ m^−2^ s^−1^, respectively), checking an increase of 75% more than the *A* values observed in seedlings with 40% WHC (<2.0 μmol CO_2_ m^−2^ s^−1^), regardless of the shading level. However, it was observed that in the period between 45 and 60 days, *A* values under 0% SH and 100% WHC increased (Figure 2A and Table 1).

Regarding *g*_s_ at 15 days, the highest values occurred in seedlings under conditions of 30% and 70% SH + 100% WHC (0.13 and 0.12 mol H_2_O m^−2^ s^−1^), with a decrease of 85% of these values in seedlings grown in 0% and 30% SH + 40% WHC (0.02 and 0.03 mol H_2_O m^−2^ s^−1^). Reduction in *g*_s_ in all cultivation conditions at 30 and 45 days was observed, with resumption after 45 days. At 60 days of evaluation, the highest value occurred in seedlings grown in 30% SH + 100% WHC, whereas the lowest values were observed in seedlings grown in 70% SH + 40% WHC in 30% SH + 70% WHC (Figure 2B).

At 15 and 60 days, the highest *A/C*_i_ values were observed in seedlings under 0% SH + 100% WHC in full sun. The lowest values occurred in seedlings grown in 70% SH + 40% WHC. At 30 and 45 days, the highest values were observed in seedlings with 30% SH + 70% WHC and 70% SH + 100% WHC. On the other hand, at the same times, seedlings grown in 0% SH + 40% WHC, 70% SH + 40% WHC, and 30% SH + 40% WHC showed lower values (Figure 2C).

The highest *E* value occurred at 15 days in seedlings grown in 0% SH + 100% WHC; at 30 and 45 days, the same seedlings were those with the lowest values. The lowest value observed at 15 days occurred in seedlings grown in 0% SH + 40% WHC, while at 30, 45, and 60 days, an increase in *E* values was observed. In general, seedlings corresponding to cultivation conditions of 30% SH + 40% WHC showed lower values at all assessment times (Figure 2D).

**Figure 3:**
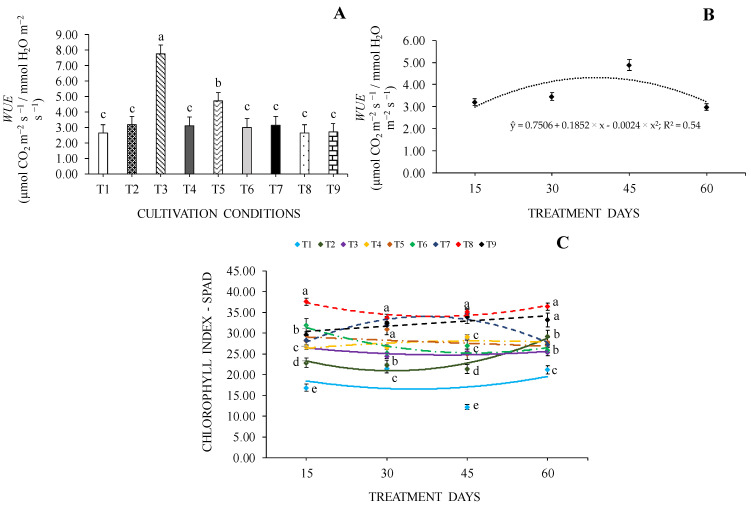
Water use efficiency—*WUE* (**A**,**B**) and chlorophyll index—SPAD (**C**) of *Cedrela fissilis* Vell. under different availability of water and light evaluated at 15, 30, 45 and 60 treatment days. T1—0% SH + 40% WHC; T2—0% SH + 70% WHC; T3—0% SH + 100% WHC; T4—30% SH + 40% WHC; T5—30% SH + 70% WHC; T6—30% SH + 100% WHC; T7—70% SH + 40% WHC; T8—70% SH +70% WHC; T9—70% SH + 100% WHC. SH—shading and WHC—water holding capacity. Equal lowercase letters between columns or markers of different colors in each evaluation period do not differ statistically according to the Scott-Knott test (*p* ≤ 0.05) ± standard error for cultivation conditions.

For *WUE*, we observed that there was no interaction between the cultivation conditions and the evaluation periods, so the data were evaluated separately. Among cultivation conditions, a 45% increase in *WUE* values was observed in seedlings grown in 0% SH + 100% WHC (Figure 3A). Regarding days of cultivation, *WUE* values were lower at 15 and 60 days and higher at 30 and 45 days (Figure 3B).

The chlorophyll index (SPAD) was influenced by the interaction between factors under study, in which the highest values were observed in seedlings at 70% SH + 70% WHC at all treatment days. At 30 and 45 days, seedlings grown in 70% SH had the highest SPAD values, and the lowest values were observed in seedlings grown in 0% SH, regardless of WHC, at the same time. Seedlings at 0% SH + 40% WHC were those with the lowest SPAD values throughout the treatment days (Figure 3C).

### 2.3. Chlorophyll a Fluorescence

**Figure 4:**
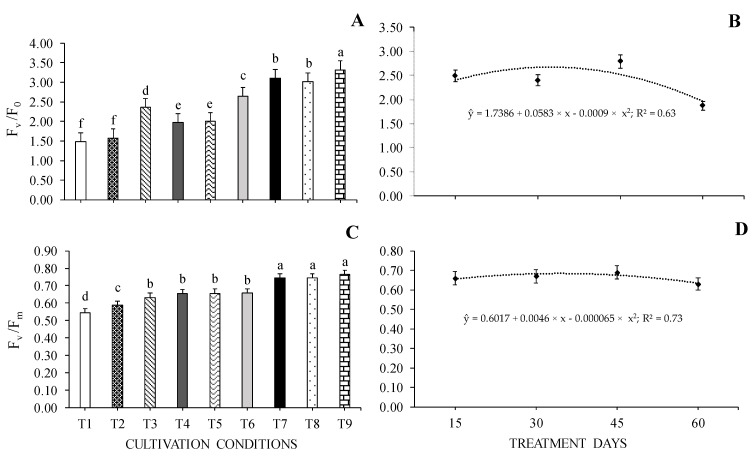
Effective quantum yield of photochemical energy conversion in PSII—F_v_/F_0_ (**A**,**B**) and potential quantum efficiency of PSII—F_v_/F_m_ (**C**,**D**) of *Cedrela fissilis* Vell. under different availability of water and light evaluated at 15, 30, 45, and 60 treatment days. T1—0% SH + 40% WHC; T2—0% SH + 70% WHC; T3—0% SH + 100% WHC; T4—30% SH + 40% WHC; T5—30% SH + 70% WHC; T6—30% SH + 100% WHC; T7—70% SH + 40% WHC; T8—70% SH +70% WHC; T9—70% SH + 100% WHC. SH—shading and WHC—water holding capacity. Equal lowercase letters between columns in each evaluation period do not differ statistically according to the Scott-Knott test (*p* ≤ 0.05) ± standard error for cultivation conditions.

There was a 50% increase in the effective quantum yield of photochemical energy conversion in PSII (F_v_/F_0_) in seedlings grown under 70% SH, regardless of WHC. The lowest values were observed for seedlings under 0% SH + 40% WHC and 0% SH + 70% WHC (Figure 4A).

Regarding the potential quantum efficiency of PSII (F_v_/F_m_), seedlings under 70% SH were those that presented the highest values regardless of WHC, with a 60% reduction in the values of this characteristic in the seedlings under 0% SH + 40% WHC (Figure 4C). Regarding values throughout the days of cultivation, the highest F_v_/F_0_ and F_v_/F_m_ values were 2.67 and 0.683 at 32 and 37 days, respectively (Figure 4B,D).

### 2.4. Relative Leaf Water Content and Growth

**Figure 5:**
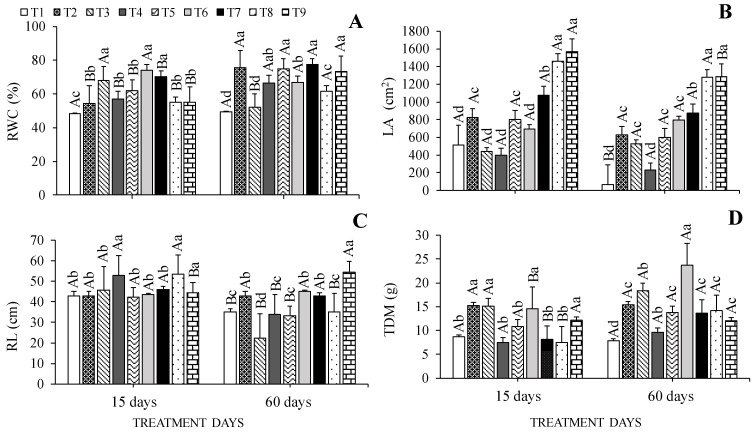
Relative water content of leaves—RWC (**A**), leaf area—LA (**B**), root length—RL (**C**), and total dry mass—TDM (**D**) of *Cedrela fissilis* Vell. under different availability of water and light evaluated at 15 and 60 treatment days. T1—0% SH + 40% WHC; T2—0% SH + 70% WHC; T3—0% SH + 100% WHC; T4—30% SH + 40% WHC; T5—30% SH + 70% WHC; T6—30% SH + 100% WHC; T7—70% SH + 40% WHC; T8—70% SH + 70% WHC; T9—70% SH + 100% WHC. SH—shading and WHC—water holding capacity. Lowercase letters compare the effect of cultivation conditions at each evaluation time using the Scott-Knott test (*p* ≤ 0.05), and uppercase letters compare the effect of days of cultivation within each cultivation condition using the F test (*p* ≤ 0.05) ± standard error.

WHC, LA, RL, and TDM values were influenced by the interaction between cultivation conditions and treatment days. Regarding relative water content (RWC), the highest values observed at 15 days occurred in seedlings grown in 0% SH + 100% WHC, being 35% higher than the values in those grown in 0% SH + 40% WHC. At 60 days, the highest values were in seedlings under 0% SH + 70% WHC, 30% SH + 70% WHC, 70% SH + 40 WHC, and 70% SH + 100% WHC, while the lowest value was observed in cedar (*Cedrela fissilis* Vell.) seedlings grown in 0% SH + 40% WHC (Figure 5A).

At 15 and 60 days, *Cedrela fissilis* Vell. seedlings submitted to the cultivation conditions of 70% SH + 70% WHC and 70% SH + 100% WHC showed the highest leaf area values. On the other hand, at 15 days, the lowest values were observed for seedlings under 0% SH + 40% WHC and 0% SH + 100% WHC and in 30% SH + 40% WHC, and at 60 days, values were observed at 0% SH + 40% WHC (Figure 5B).

The highest root length values at 15 days were observed for seedlings submitted to 30 and 70% SH, both with 70% WHC; the seedlings from the other treatments at the same time did not differ from each other, corresponding to the lowest values. At 60 days, seedlings under 70% SH + 100% WHC showed a 50% increase in RL values compared to seedlings under 0% SH + 100% WHC (Figure 5C).

Regarding total dry mass (TDM), the highest values were observed for seedlings at 0% SH + 70 and 100% WHC and in those at 30% SH + 100% WHC at 15 days. However, in the same period, seedlings under 40% WHC had lower values, regardless of shading level. At 60 days, the highest and lowest TDM values occurred in seedlings grown in 30% SH + 100% WHC and 0% SH + 40% WHC, respectively (Figure 5D).

### 2.5. Phenotypic Plasticity

Seedlings grown in 30% SH corresponded to the highest phenotypic plasticity index values for *A* and TDM. The highest PPI values for F_v_/F_m_ and LA were observed for seedlings grown in full sun. Seedlings grown in the 70% SH environment presented lower PPI values for *A*, F_v_/F_m_, LA, and TDM (Table 2).

## 3. Discussion

*Cedrela fissilis* Vell. seedlings are sensitive to water deficit, here represented by 40% WHC, as they showed reduced photosynthetic metabolism and growth, and when cultivated in full sun, these stressful effects were enhanced, acting in a synergistic way. However, it was observed that shading, especially 70% SH, mitigates the stressful effect of water deficit.

Changes in the soil-water regime can influence the morphological and physiological responses of plants [16,17]. Reductions in *A*, *g*_s_, and *E* values in seedlings grown in 40% WHC were observed, regardless of shading levels. However, the full sun environment further accentuated stress due to water deficit, because under this cultivation condition, in addition to the fact that soil water status is lower, there is greater evaporation of water from the substrate and exposure to higher temperatures, a fact contrary to the shaded environment, which reduces the evapotranspiration intensity, contributing to the maintenance of RWC in cells and their turgidity. Similarly, ref. [18] observed that shading in the cultivation of *Campomanesia xanthocarpa* (Mart.) O. Berg minimized the negative effects of water deficit on gas exchange, and the authors associate this response to the fact that shading allowed less water loss through the substrate, which favored stomatal opening for a longer time and resulted in greater CO_2_ assimilation.

One of the first responses to stress due to water scarcity is stomatal closure, which is mediated by a complex signaling network involving abscisic acid [19], as a mechanism to prevent excessive water loss through leaf transpiration and maintain water potential [7,20,21], a fact observed in seedlings at 30 days under the 0% SH + 40% WHC treatment, where radiation in full sun reached values of 2038 μmol m^−2^ s^−1^, this high radiation combined with low water availability resulted in a reduction in *g*_s_. Likewise, [7] found that *C. fissilis* showed greater transpiration rate control as a mechanism to reduce water loss.

At the same time that stomatal closure acts in water regulation, this mechanism limits the efficiency of the biochemical step of photosynthesis in *C. fissilis* seedlings. According to [22], this reduction is due to the fact that there is a decrease in mesophyll conductance and CO_2_ fixation in the chloroplast due to the lower carboxylation efficiency. On the other hand, although there is a reduction in gs after 30 days of exposure to stress, there is an increase in conductance values in seedlings under 30% SH + 40% WHC and 0% SH + 40% WHC, indicating that as a survival mechanism in the face of environmental variations, plants regulate the stomatal openings over time so that there is less water loss without harming the entry of CO_2_. From 45 days onwards, *g*s and *A* values increased; precisely during this period the seedlings were receiving radiation in full sun of 1938 μmol m^−2^ s^−1^, suggesting that the species presents a better performance in this condition. Therefore, it is emphasized that the species presents adjustment strategies that ensure its phenotypic plasticity and potential for ecological resilience.

Another factor that may have contributed to the 0% SH environment to accentuate the reduction in photosynthetic metabolism, especially in seedlings under 40% WHC, was the increase in leaf temperature that occurred in this environment. Studies have reported that high temperatures directly impact the photosynthetic enzymes involved in carbon assimilation, being harmful to the proper functioning of photosynthetic processes [23]. As shading increases, a reduction in leaf temperature is observed, demonstrating that shading mitigated the negative effects caused by the increase in temperature.

The lower water availability, represented here by 40% WHC, combined with the highest light intensity during the evaluation periods (0% SH) reduced the chlorophyll index and photochemical activities in the photosystem II of *C. fissilis* seedlings in all evaluation periods. Excessive light energy induces photoinhibition of photosystem II (PSII) and causes photodamage to the photosynthetic system [24], also associated with reduction in ATP as a result of the lower H+ production due to water deficit [25], reducing photosynthetic carbon gain. The full sun condition associated with lower water availability on treatment days caused a decrease in these factors, indicating that there was a decline in the PSII function and that photosynthetic units and electron transfer processes linked to the membrane were affected under this stress [26].

Under the same cultivation conditions mentioned above, seedlings presented lower leaf area values, which, although considered a strategy found by plants to minimize water loss through evapotranspiration, reduces the photosynthetically active area. Furthermore, this response is associated with a reduction in turgor, since under the same cultivation condition, lower WHC was also observed.

Conversely, the full sun environment (0% SH) combined with high water availability (100% WHC) provided *C. fissilis* seedlings with higher *A*, *A/C*_i_, *E,* and RWC values, considering that in the first evaluation (15 days) the radiation was around 1159 μmol m^−2^ s^−1^, which shows us that if the species is cultivated at this level of shading, it is necessary to increase the water regime to meet energy costs and alleviate the negative effect of high light intensity.

The fact that the 0% SH + 100% WHC condition provided higher *A/C*_i_ values on treatment days 15 and 60, where radiation was 1159 and 1212 μmol m^−2^ s^−1^ respectively, can be justified by studies that report that plants grown under adequate irradiance, in this case associated with high water availability, present an increase in Rubisco content and activity to optimally use more absorbed light energy [27,28,29].

However, on treatment days 30 and 45, seedlings under 0% SH + 100% WHC showed a decrease in the values of *A*, *g*_s_, *A/C*_i,_ and *E*, at which time there was an increase in radiation radiation received by the seedlings. Between 45 and 60 days, the values of these variables increased, suggesting that the 0% SH condition associated with greater water availability was initially beneficial for seedlings, but the high PAR caused stress; however, seedlings adapted to this condition over time, recovering their gas exchange values.

What may have helped with this response was the fact that under this cultivation condition (0% SH + 100% WHC), seedlings presented higher *WUE* values. The *WUE* is defined as the amount of carbon assimilated as biomass per unit of water used by the plant, with a lower transpiration coefficient [30]. In general, *WUE* was higher at 38 days, coinciding with the period in which gas exchange decreased for *C. fissilis* seedlings grown in full sun with high water availability. *WUE* is an important mechanism of physiological adaptation [31], and the increase in this factor led to better use of available resources.

The species’ lower sensitivity to exposure to full sun in the first evaluation can be attributed to the fact that on that day of treatment the radiation received by the seedlings was not as intense as in the other periods and therefore may have been insufficient to cause stress responses, but over time and with variations, the photosynthetic metabolism reduced, especially under 40% WHC. However, it can also be attributed to the physiological and morphological properties of its leaves, which are well adapted to greater irradiance. Pioneer or early secondary species tend to have greater capacity to increase water use efficiency under more energetic luminosity [32].

The increase in the chlorophyll index in shaded seedlings, especially under 70% SH, regardless of water regime, can be considered a compensatory mechanism. This is because the increase in chlorophyll content per area unit increases the absorption of photosynthetically active radiation to increase the efficiency of CO_2_ assimilation [32,33,34,35].

Most plants under shade invest more in the synthesis and maintenance of structures that capture light than those in full sun conditions [36]. Plants with greater tolerance to reduced light are plastic, as they can control light capture variables, such as leaf size and chlorophyll content. In this sense, higher leaf area values for *C. fissilis* in 70% shading were also observed on the different treatment days. Increasing leaf area is a strategy of the species to optimize the use of light, increasing the surface for capturing photons to maintain photosynthetic processes [37,38].

The increase in the shading level resulted in higher F_v_/F_0_ and F_v_/F_m_ values in *C. fissilis* seedlings, regardless of water regimes, which may be related to greater potential for use and optimization of available light energy [39]. The seedlings grown in 0% SH having presented a reduction in the values of F_v_/F_0_ and F_v_/F_m_ may be related to the fact that in this environment an increase in leaf temperature was observed. High temperatures can cause physiological disorders in plants; for example, photosystem I and photosystem II are negatively affected [40].

However, shading can improve the light energy use rate [41] and, in this case, can mitigate the damage caused to the photosynthetic apparatus, providing conditions that would reduce the leaf temperature of seedlings. Plant plasticity helps to improve the use of photosynthetic energy under greater light availability, and *C. fissilis* seedlings grown in 0% SH, even with radiation variations throughout the days of treatment, showed higher PPI for F_v_/F_m_ as a mechanism for adjusting the photosynthetic apparatus in response to unfavorable conditions.

*C. fissilis* seedlings that presented the highest total dry mass values were those grown in moderate shading (30% shading) combined with 100% WHC. This condition provided seedlings with adjustments in gas exchange throughout the evaluation periods for carrying out the photosynthetic process, such as the regulation of the ideal stomatal opening, providing the entry of CO_2_, as well as higher transpiration levels and Rubisco carboxylation activity.

*C. fissilis* is considered a species with high light demand for growth [42]. Even though this species is classified as pioneer or initial secondary, excess light under full sun conditions, given the variations in radiation received over time, caused a reduction in the photosynthetic metabolism of seedlings, which reflected in less investment in seedling growth, an accentuated response under 40% WHC. Although plants increase *A* values over days of treatment, reductions that occurred before seedlings adapted to the condition caused plants to invest their energy in survival to the detriment of growth. In the present study, it was found that the best seedling performance occurred under moderate shading combined with greater water availability.

Moderate shading associated with greater water availability resulted in greater production of photoassimilates for investment in greater dry mass in *C. fissilis*. Ref. [7] found that the survival and growth of *C. fissilis* were associated with adequate water availability (70% WHC and 100% WHC). This is because low water availability directly affects photosynthesis and consequently organic matter accumulation [43], as observed in this study with *C. fissilis.*

*C. fissilis* seedlings showed higher phenotypic plasticity rates for *A*, demonstrating that under different light conditions, this species adjusts in order to maintain its photosynthetic activity and survival. Some studies have reported that leaf characteristics are highly plastic, although phenotypic plasticity measures are strongly correlated with the context and are not comparable between different studies, gradients, and species [44,45]. In the full sun environment, *C. fissilis* showed greater morphological adjustments in the leaf area and physiological adjustments in F_v_/F_m_, reinforcing that this cultivation condition was stressful, especially when associated with 40% WHC, a result confirmed by the highest PPI values.

Seedlings showed few adjustments in the 70% shading condition, which leads us to believe that for the factors evaluated under this environmental condition, plants did not need many adjustments to guarantee their survival. However, 70% shading did not correspond to the condition in which seedlings best expressed their potential.

Based on our results, *C. fissilis* showed desirable characteristics for forest restoration and reforestation. It is possible to verify that the species presents plasticity to contrasting light environments, but that in the seedling phase, its best morphophysiological performance is under moderate shading, reinforcing its classification as initial secondary. As practical implications to be implemented in nurseries, if the cultivation is in full sun, for example in open areas, the ideal would be to use a water regime with greater availability to supply its photosynthetic metabolism, while if implemented in areas with low water status or subject to temporary drought, shaded cultivation is a strategy to mitigate stressful effects.

Given the above, we suggest future research exploring the behavior of this species under these conditions for a longer period of time, including evaluations after transplanting the seedlings to the final location where the tree will develop.

## 4. Material and Methods

### 4.1. Experiment Location and Seedling Production

The experiment was conducted at the Federal University of Grande Dourados—UFGD, implemented in different light conditions of the Faculty of Agricultural Sciences (22°11′43.7″ S and 54°56′08.5″ W, 452 m a.s.l.), in the city of Dourados—MS, Brazil.

*C. fissilis* seedlings were produced from ripe fruits collected in a remaining Cerrado area (Authorization for Access and Shipment of Genetic Heritage Component Sample No. 010220/2015-1–CNPq/CGEN/MMA), in the state of Mato Grosso do Sul. The collection of fruits and seeds was carried out during periods of dispersal, and processing was manually performed so that seeds were extracted and selected.

Sowing took place on 2 August 2022 in tubes with a volume of 280 cm^3^ filled with substrate consisting of Distroferric Red Latosol + commercial substrate (Bioplant^®^) (1:1, *v*/*v*). Tubes were placed on benches in a nursery environment protected with black nylon screen (Sombrite^®^) with 30% shading. Full emergence occurred 90 days after sowing (DAS) on 12 November 2022.

Transplanting into pots was carried out when seedlings reached an average height of 8.0 cm. Seedlings were grown in 8 kg plastic pots filled with substrate composed of Distroferric Red Latosol + coarse sand (3:1, *v*/*v*), with two plants each on 15 December 2022.

### 4.2. Shading Level and Water Regimes

**Figure 6:**
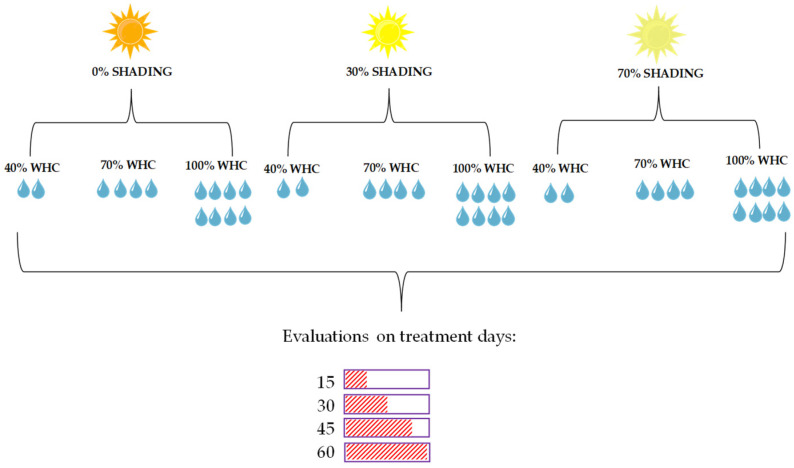
Experimental design exemplifying the origin of the 9 treatments is the interaction between the three levels of shading with the three water retention capacities and the four periods in which the seedlings of all treatments were evaluated.

After transplanting, seedlings were submitted to acclimatization for 45 days at each shading level—SH (0, 30, and 70%). After this period, seedlings were submitted to three water regimes based on the water holding capacity (WHC) in the substrate, constituting nine cultivation conditions: T1—0% SH + 40% WHC; T2—0% SH + 70% WHC; T3—0% SH + 100% WHC; T4—30% SH + 40% WHC; T5—30% SH + 70% WHC; T6—30% SH + 100% WHC; T7—70% SH + 40% WHC; T8—70% SH + 70% WHC; T9—70% SH + 100% WHC. The experimental design used was completely randomized in a factorial scheme with 9 cultivation conditions (WHC and shading combination) × 4 evaluation times, with four replicates (Figure 6). The experimental unit consisted of one pot with two seedlings each. These different light gradients and water regimes were chosen based on results obtained in previous work [6,18], where tree species were responsive.

The different water regimes and shading levels were applied from implementation until the last evaluation (treatment days). In the different light conditions, a structure with a top and side cover made of transparent plastic was inserted to protect against possible precipitations in the experiment. Shading levels of 30 and 70% were obtained through the use of black nylon screens (Sombrite^®^), in which photosynthetically active radiation and leaf temperature values were recorded on a portable photosynthesis meter LCIPro—SD (IRGA) ADC BioScientific Ltd. Global House, Geddings Road, Hoddesdon, Herts EN11 0NT, UK and can be found in Table 3.

**Figure 7:**
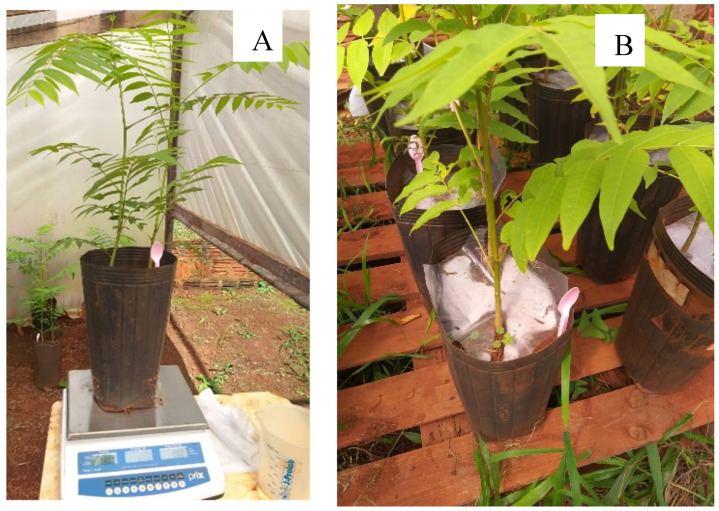
Maintenance of the WHC, with the aid of scales on *Cedrella fissilis* Vell. seedlings (**A**). Pots with plastic protection used to reduce soil water evapotranspiration (**B**).

The water holding capacity of the substrate was determined according to the methodology of [46], in which 100% WHC was obtained through the water content retained after draining, and the others were calculated using the simple rule of three as a function of weight. Pots were weighed on a precision scale (0.001 g) (Figure 7), and individual irrigation was carried out daily, adding enough water to reach the pre-established weight for each cultivation condition. The substrate of each pot was covered with plastic to prevent water evaporation from the soil.

### 4.3. Evaluations

Gas exchange, chlorophyll a fluorescence, and chlorophyll index characteristics were evaluated at 15, 30, 45, and 60 days of cultivation, while those of relative water content and growth were evaluated at 15 and 60 days.

Gas exchange, chlorophyll a fluorescence, and chlorophyll index determination were carried out between 8 and 11 a.m. on the same fully expanded and previously marked leaves, so that all measurements were always carried out on the same plants.

#### 4.3.1. *Gas Exchange and Chlorophyll Index*

The net photosynthetic rate—*A* (µmol CO_2_ m^−2^ s^−1^), stomatal conductance—*g*_s_ (mol H_2_O m^−2^ s^−1^) and transpiration rate—*E* (mmol H_2_O m^−2^ s^−1^) were quantified with a portable photosynthesis meter (LCIPro—SD ADC BioScientific Ltd.). From these data, the Rubisco carboxylation efficiencies—*A/C*_i_ (µmol mol CO_2_ m^−2^ s^−1^) and water use efficiency—*WUE*—A/E (µmol CO_2_/mmol H_2_O) were calculated.

The chlorophyll index—SPAD was obtained using a portable chlorophyll meter (model SPAD-502, Minolta Camera Co., Ltd. Chuo-ku, Osaka 541, Japan) and carried out on the median limbs of the same leaves as the gas exchange and fluorescence assessments. It is a non-destructive method, not damaging the leaf.

#### 4.3.2. *Chlorophyll a Fluorescence*

The potential quantum efficiency of PSII—F_v_/F_m_ and effective quantum yield of photochemical energy conversion in PSII—F_v_/F_0_ were determined using portable fluorometer model OS-30p (Opti-Sciences Chlorophyll Fluorometer, Hudson, NY, USA). Leaves were submitted to a period of 30 min of dark adaptation with the aid of adapter clips so that all reaction centers in this leaf region reached the “open” condition, that is, complete oxidation of the electron transport photosynthetic system [47].

#### 4.3.3. *Relative Leaf Water Content and Growth*

The relative water content of leaves—RWC—was calculated using the methodology of [48] according to the formula:

$$RWC=\left(\left(FW-DW\right)÷\left(TW-DW\right)\right)\times 100$$

where: FW = Fresh weight; DW = Dry weight; TW = Turgid weight.

The root length (RL) was measured with a graduated ruler, and leaf area—LA (cm^2^)—was evaluated using the ImageJ software V. 1.8.0. The materials were placed in an oven with forced air circulation at 60 ± 5 °C for 72 h and weighed on a decimal precision scale (0.0001 g).

#### 4.3.4. *Phenotypic Plasticity Index (PPI)*

The PPI for *A*, F_v_/F_m_, LA, and TDM was calculated from data obtained after 60 days of cultivation according to the methodology proposed by [49]. PPI was calculated considering the highest and lowest values of these characteristics, calculating between the highest and lowest value between water regimes for each shading level.

### 4.4. Data Analysis

Data were submitted to analysis of variance (ANOVA), and when significant by the F test (*p* ≤ 0.05), means were compared by the Scott-Knott test (*p* ≤ 0.05) for the different cultivation conditions. Data analyzed at 15 and 60 days were compared by the F test (*p* ≤ 0.05) and at 15, 30, 45, and 60 days by regression analysis (*p* ≤ 0.05), using the SISVAR 5.3 statistical software [50]. The averages as a function of time will be considered adjusted only when the linear or quadratic models are significant. PPI results were presented in a descriptive manner without applying statistical analysis.

## 5. Conclusions

The availability of 40% water holding capacity was detrimental to gas exchange and caused a reduction in the chlorophyll index, regardless of exposure time to different radiations, and consequently affected the growth of *C. fissilis*, especially when combined with a full-sun environment.

Seedlings grown in 70% shade presented strategies such as adaptation mechanisms to survive, demonstrating that this species has physiological plasticity, and this condition helped to mitigate the stressful effect of water deficit in *C. fissilis*.

The condition that provided better gas exchange performance and greater total dry mass accumulation for *C. fissilis* seedlings was 30% shading combined with 100% WHC. *C. fissilis* seedlings showed physiological plasticity and resilience to survive under different water and light conditions.

## Figures and Tables

**Table 1:** Regression equations of the effect of evaluation time for photosynthetic rate (*A*), stomatal conductance—(*g*_s_), Rubisco carboxylation efficiencies (*A/C*_i_), transpiration rate—(*E*), and chlorophyll index (SPAD) in each cultivation condition in *Cedrela fissilis* Vell. seedlings. T1—0% SH + 40% WHC; T2—0% SH + 70% WHC; T3—0% SH + 100% WHC; T4—30% SH + 40% WHC; T5—30% SH + 70% WHC; T6—30% SH + 100% WHC; T7—70% SH + 40% WHC; T8—70% SH +70% WHC; T9—70% SH + 100% WHC. SH—shading and WHC—water holding capacity. Ŷ = calculated value of the response variable; R^2^ = determination coefficient. No adjustment = no liner or quadratic.

	Characteristics Evaluated			
	*A* (µmol CO_2_ m^−2^ s^−1^)	*g*_s_ (mol H_2_O m^−2^ s^−1^)		*A/C*_i_ (µmol mol CO_2_ m^−2^ s^−1^/µmol m^−2^ s^−1^)
T1	ŷ = 4.8925 − 0.1979 × x + 0.0029 × x^2^; R^2^ = 0.96	ŷ = 0.0333 − 0.0017 × x + 0.000037 × x^2^; R^2^ = 0.97		No adjustment
T2	No adjustment	ŷ = 0.1108 − 0. 0051 × x + 0.000078 × x^2^ R^2^ = 0.96		ŷ = 0.0091 + 0.00076 × x − 0.000010 × x^2^; R^2^ = 0.88
T3	ŷ = 13.8925 − 0.5310 × x + 0.0071 × x^2^; R^2^ = 0.70	No adjustment		ŷ = 0.0492 − 0.00195 × x + 0.000028 × x^2^; R^2^ = 0.89
T4	ŷ = 5.825 − 0.2688x + 0.0039 × x^2^; R^2^ = 0.94	ŷ = 0.0525 − 0.0030 × x + 0.000048 × x^2^; R^2^ = 0.95		ŷ = 0.0289 − 0.0013 × x + 0.000018 × x^2^; R^2^ = 0.93
T5	No adjustment	No adjustment		No adjustment
T6	ŷ= −0.3825 + 0.3827 × x − 0.0042 × x^2^; R^2^ = 0.93	ŷ = 0.2658 − 0.0114 × x + 0.000152 × x^2^; R^2^ = 0.99		ŷ = −0.0152 + 0.0024 × x − 0.000029 × x^2^; R^2^ = 0.98
T7	No adjustment	ŷ = 0.0083 + 0.0026 × x − 0.000044 × x^2^; R^2^ = 0.72		ŷ = 0.0032 + 0.000099 × x; R^2^ = 0.73
T8	ŷ= −1.3425 + 0.3626 × x − 0.0041 × x^2^; R^2^ = 0.98	No adjustment		ŷ = −0.0072 + 0.0011 × x − 0.000014 × x^2^; R^2^ = 0.86
T9	ŷ = −1.3275 + 0.3303 × x − 0.0037 × x^2^; R^2^ = 0.65	ŷ = 0.2283 − 0.0086 × x + 0.000096 × x^2^; R^2^ = 0.99		No adjustment
	*E* (mmol H_2_O m^−2^ s^−1^)		Chlorophyll index (SPAD)	
T1	ŷ = −0.0033 + 0.0318 × x; R^2^ = 0.78		No adjustment	
T2	ŷ = 0.7100 + 0.0264 × x; R^2^ = 0.80		ŷ = 29.8666 − 0.5777 × x + 0.0093 × x^2^; R^2^ = 0.88	
T3	ŷ = 6.330 − 0.3102 × x + 0.0040 × x^2^; R^2^ = 0.96		No adjustment	
T4	ŷ = 1.2658 − 0.0499 × x + 0.00083 × x^2^; R^2^ = 0.83		No adjustment	
T5	No adjustment		No adjustment	
T6	ŷ = −0.1050 + 0.1275 × x − 0.00135 × x^2^; R^2^ = 0.71		ŷ = 38.6916 − 0.5836 × x + 0.0063 × x^2^; R^2^ = 0.76	
T7	No adjustment		ŷ = 16.8666 + 0.9144 × x − 0.0122 × x^2^; R^2^ = 0.87	
T8	No adjustment		ŷ = 43.0416 − 0.4765 × x + 0.0061 × x^2^; R^2^ = 0.79	
T9	No adjustment		ŷ = 29.200 + 0.0824 × x; R^2^ = 0.69	

**Table 2:** Phenotypic plasticity index (PPI) for photosynthetic rate (*A*), potential quantum efficiency of PSII (F_v_/F_m_), leaf area (LA), and total dry mass (TDM) of *Cedrela fissilis* Vell. plants grown in different shading levels at 60 days.

Shading Level	PPI (0.00 to 1.00)			
	*A*	F_v_/F_m_	LA	TDM
0%	0.49	0.16	0.88	0.42
30%	0.54	0.04	0.70	0.48
70%	0.45	0.02	0.30	0.38

**Table 3:** Average photosynthetically active radiation and leaf temperature values at each shading level at different days of cultivation in the cultivation of *Cedrela fissilis* Vell. seedlings.

Evaluation Period	Shading Level	PAR	Leaf T. (°C)
Days		(μmol m^−2^ s^−1^)	(°C)
15	0%	1159	38.0
	30%	810	33.0
	70%	347	32.0
30	0%	2038	33.0
	30%	1426	31.7
	70%	611	32.0
45	0%	1934	42.0
	30%	1353	36.8
	70%	580	36.1
60	0%	1212	40.3
	30%	848	38.2
	70%	363	31.5

## Data Availability

The data presented in this study are available in the graphs and tables provided in the manuscript.
